# Corneal Endothelial Pathology and Thickening in Early Diabetes Mellitus

**DOI:** 10.22336/rjo.2026.35

**Published:** 2026

**Authors:** Konstantin Gushansky, Anry Pitchkhadze

**Affiliations:** 1 Ophthalmology Department, Soroka University Medical Center, Ben-Gurion University of the Negev, Be’er Sheva, Israel

**Keywords:** corneal endothelium, endothelial cell density, central corneal thickness, coefficient of variation, Type 2 Diabetes Mellitus, AGE = Advanced glycation end-product, ANOVA = Analysis of variance, CCT = Central corneal thickness, CoV = Coefficient of variation, ECD = Endothelial cell density, ETDRS = Early Treatment Diabetic Retinopathy Study, HbA1c = Glycated hemoglobin, IOP = Intraocular pressure, T2DM = Type 2 diabetes mellitus

## Abstract

**Purpose:**

To compare endothelial cell properties and central corneal thickness (CCT) between nondiabetic individuals and patients with early type 2 diabetes mellitus (T2DM) with good glycemic control.

**Methods:**

This retrospective case-control study included patients diagnosed with T2DM within the past 10 years and nondiabetic individuals. All participants underwent comprehensive ocular examination and specular microscopy to measure endothelial cell density (ECD), central corneal thickness (CCT), and coefficient of variation (CV). Intergroup differences were analyzed statistically.

**Results:**

A total of 312 eyes from 180 patients were included: 118 eyes in the diabetic group and 194 in the nondiabetic group. There were no significant differences in age (p = 0.08) or sex distribution (p = 0.49) between groups. The diabetic group had a significantly lower mean ECD compared with controls (2467.8 ± 334.5 vs. 2553.5 ± 289.2; p = 0.028) and a significantly higher CCT (558.8 ± 48.8 vs. 547.1 ± 44.6; p = 0.034). No significant difference was observed in the coefficient of variation.

**Discussion:**

These findings suggest that corneal endothelial alterations may occur early in the course of type 2 diabetes mellitus, even in patients with good glycemic control and short disease duration. The observed reduction in endothelial cell density alongside increased central corneal thickness may reflect subclinical endothelial dysfunction or early metabolic effects on corneal hydration regulation, preceding overt clinical corneal disease.

**Conclusions:**

Early T2DM is associated with reduced ECD and increased CCT compared with nondiabetic individuals, despite good glycemic control. These results indicate that diabetes may affect corneal endothelial structure and function at an early stage.

## Introduction

The corneal endothelium plays a vital role in preserving corneal thickness, hydration, and transparency. It consists of hexagonal cells that lack mitotic capability *in vivo* [1]. Endothelial cell density can decline due to various factors, including normal aging and cataract surgery. To compensate, the remaining endothelial cells expand and migrate, often resulting in irregular cell shapes. If the cell density falls below a critical threshold, bullous keratopathy may develop, leading to significant vision loss [2].

Diabetes mellitus has been associated with endothelial cell loss and morphological abnormalities, including increased polymegathism and pleomorphism [3-5]. Moreover, diabetic patients typically exhibit higher central corneal thickness (CCT) compared to non-diabetic individuals [4,6]. Maintaining effective glycemic control in diabetic patients is crucial for managing surgical risks, as hyperglycemia alone can induce endothelial changes significant enough to elevate a routine procedure to a high-risk surgery [7].

A pertinent question is whether corneal endothelial pathology and increased central corneal thickness (CCT) emerge in the early stages of well-controlled diabetes or are hallmarks of advanced or poorly managed disease. To our knowledge, no studies utilizing modern specular microscopy have investigated this issue. Therefore, we evaluated and compared endothelial characteristics and CCT between nondiabetic individuals and patients recently diagnosed with type 2 diabetes who demonstrated good glycemic control.

## Methods

This study adhered to the principles of the Declaration of Helsinki and received approval from the institutional review board.

### Study Design

This retrospective case-control study was conducted at the Ophthalmology Department of Soroka University Medical Center, Beer Sheva, Israel.

### Patients

Key inclusion criteria included adult patients aged 50 to 90 years with a diagnosis of type 2 diabetes mellitus (T2DM) based on WHO criteria [8] made within the preceding 10 years.

The control group consisted of nondiabetic patients matched for age and gender distribution. Exclusion criteria included a history of prior intraocular or ocular surface surgery, contact lens use, trauma, intraocular inflammation, corneal infections, or diagnosed corneal pathology (except dry eye). Patients were also excluded if their diabetes status was undocumented in medical records. All participants underwent a comprehensive ocular evaluation, including a review of medical history, slit-lamp examination of the anterior segment, fundus examination, and specular microscopy. Diabetic retinopathy was classified according to the Early Treatment Diabetic Retinopathy Study (ETDRS) standardization protocols.

### *Assessment*

Specular microscopy was performed using the SP-02 specular noncontact microscope (C.S.O., Scandicci, Florence, Italy), a device widely employed in both clinical practice and research [9]. High-resolution images of the central corneal endothelium were obtained, with the accompanying software automatically identifying contiguous endothelial cells for analysis using the corner method. To minimize sampling error, patients with fewer than 125 analyzable cells per image were excluded from the analysis [10]. The following parameters were calculated: endothelial cell density (ECD), central corneal thickness (CCT), and the coefficient of variation (CV) in cell size. Pleomorphism—defined as the percentage of hexagonal cells—was also assessed as a measure of variation in cell shape.

**Fig. 1:**
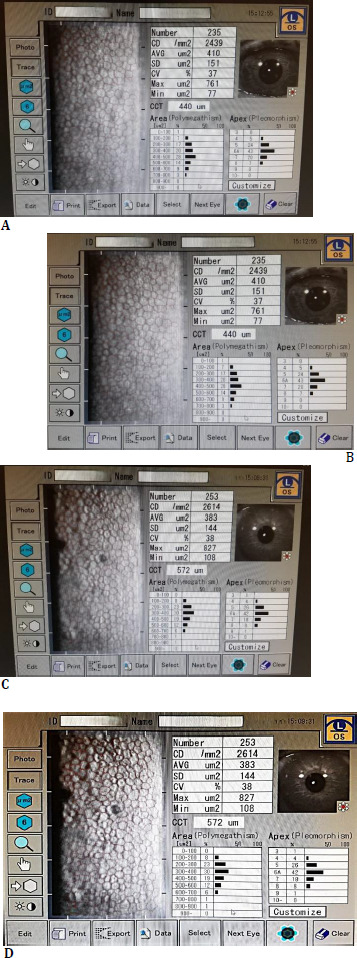
A-D. The images demonstrate automated analysis of the central corneal endothelium using the corner method. The software identifies contiguous endothelial cells to calculate endothelial cell density (ECD), central corneal thickness (CCT), and coefficient of variation (CoV) in cell size. Pleomorphism, defined as the percentage of hexagonal cells, is also displayed as a measure of endothelial cell shape variability

Fig. 1 shows a representative specular microscopy output from the SP-02 noncontact specular microscope (C.S.O., Scandicci, Florence, Italy).

### *Statistical Analysis*

Statistical analyses were performed using the Statistical Package for the Social Sciences (SPSS) software (version 17.0; SPSS, Inc., Chicago, IL). Pearson’s correlation was used for parametric data, while Spearman’s correlation was applied to non-parametric data. Mean comparisons were conducted using the t-test and one-way ANOVA. To address potential confounders, such as age, gender, and intrapersonal data variability, a multiple regression analysis was performed using the non-standardized B coefficient [11]. A p-value of ≤ 0.05 was considered statistically significant.

## Results

The study included 312 eyes from 180 patients: 118 eyes in the diabetic group and 194 in the control group (nondiabetic patients). The overall mean age was 70.9 ± 7.8 years. Both groups demonstrated comparable age and sex distributions, with no statistically significant differences observed (p = 0.49 and p = 0.08, respectively). Detailed demographic characteristics of the study population are presented in Table 1.

**Table 1:** Baseline Demographic Data of the 180 Study Participants

Parameter		Diabetic	Non-diabetic	p value
Gender	Male	37	31	0.49
	Female	55	57	
Age		71.9 ± 7.7	70.3 ± 7.9	0.08

The Chi-square test and unpaired t-test were used to detect statistically significant differences between groups in gender and age, respectively.

The study group had a mean diabetes duration of 7.8 years and an HbA1c level of 7.2%. Additionally, the majority of patients in the study group did not have diabetic retinopathy. In the control group, the mean fasting glucose level was 89.7 ± 12.45 mg/dL. Details regarding diabetes duration, glycemic control indices, and retinopathy data are summarized in Table 2.

**Table 2:** Diabetes Characteristics and Retinopathy Data of the 180 Study Participants

Parameter	Diabetic	Non-diabetic	p value
Diabetes Duration (Mean ± SD)	7.8 ± 6.2 years	-	
HbA1C % (Mean ± SD)	7.2% ± 1.2%	-	
Fasting Glucose (Mean ± SD)	135 ± 47.5 mg/dl	89.7±12.45 mg/dl	<0.0001
Diabetic Retinopathy	None 88%	-	
	Non-Proliferative 9%		
	Proliferative 3%		

A paired t-test was conducted to detect statistically significant differences in fasting glucose levels between groups. SD, standard deviation. P-values less than 0.05 are considered statistically significant and are presented in bold.

Pearson correlation analysis revealed significant correlations among specular microscopy findings, diabetes duration, and fasting glucose and hemoglobin A1c levels. Statistically significant differences were also observed between the study groups. Raw data were adjusted for various factors, including age, gender, eye laterality (right vs. left), intraocular pressure (IOP), retinopathy, and whether one or both eyes were evaluated. A summary of the multiple linear regression analysis is presented in Table 3.

**Table 3:** Summary of Multiple Linear Regression Analysis for 312 Eyes

Parameter	Diabetes Diagnosis		Diabetes Duration	
	B Regression Value	p value	B Regression Value	p value
ECD	-79.1	0.03	4.9	0.39
CCT (µm)	11.34	0.03	1.51	0.05
Cell Area (µm2)	15.1	0.021	1.27	0.29
CoV (%)	1.1	0.2	0.14	0.9
Hexa (%)	-0.34	0.68	-0.59	0.612

ECD: Endothelial cell density (mean ± standard deviation), CCT: Central corneal thickness, CoV: Coefficient of variation, Hexa: Percentage of hexagonal cells.

The adjusted mean endothelial cell density was significantly lower in the diabetic group than in the control group (2467.8 ± 334.5 vs. 2553.5 ± 289.2; p = 0.028). Additionally, adjusted central corneal thickness and cell area were significantly greater in the diabetic group. A borderline association was observed between adjusted central corneal thickness and diabetes duration. However, no statistically significant differences were found in the coefficient of variation or the percentage of hexagonal cells. HbA1c and fasting glucose levels were not associated with any of the assessed parameters. Details of the corneal and endothelial indices are provided in Table 4.

**Table 4:** Adjusted Corneal and Endothelial Characteristics for 312 Eyes

Parameter	Diabetic	Non-diabetic	p value *
ECD	2467.8 ± 334.5	2553.5 ± 289.2	0.028
CCT (µm)	558.8 ± 48.8	547.1 ± 44.6	0.034
Cell Area (µm2)	412.9 ± 68	396.7 ± 46.6	0.021
CoV (%)	42.9 ± 7.3	41.8 ± 7.3	0.198
Hexa (%)	49.3 ± 7.0	49.6 ± 7.0	0.678

ECD: Endothelial cell density (mean ± standard deviation), CCT: Central corneal thickness, CoV: Coefficient of variation, Hexa: Percentage of hexagonal cells. * A paired t-test was performed to detect statistically significant differences between groups.

## Discussion

Corneal endothelial cells in diabetic patients exhibit both quantitative and qualitative abnormalities. Previous studies have consistently reported reduced cell density in individuals with diabetes [5,12]. Moreover, diabetic patients show distinct morphological changes compared to non-diabetic individuals, including increased central corneal thickness [4,7,1,2], elevated coefficient of variation [4], and a lower percentage of hexagonal cells [1].

Several biochemical and histologic mechanisms may contribute to these findings. Biochemically, four key processes occur in diabetic conditions: increased polyol pathway flux, activation of protein kinase C, elevated hexosamine pathway flux, and the formation of advanced glycation end-products (AGEs) [13]. Among these, AGEs are the most thoroughly understood. Prolonged hyperglycemia triggers a series of non-enzymatic reactions between glucose and proteins, leading to the formation of AGEs [14]. These compounds adversely affect the corneal endothelium through two mechanisms: intracellular or extracellular accumulation [1,5] and activation of cell membrane receptors, which results in the production of harmful cytokines [1,6]. AGEs have been linked to endothelial dysfunction in diabetes, including conditions such as Fuchs’ dystrophy [17,18]. Additionally, AGEs have been identified in Descemet’s membrane, potentially contributing to abnormal endothelial adhesion and subsequent alterations in endothelial shape and function [19].

Histologically, corneal deposits and thickening of Descemet’s membrane have been reported in diabetic patients [20]. While most studies suggest that poorly controlled diabetic patients exhibit both qualitative and quantitative alterations in the corneal endothelium [2,1], there remains an ongoing debate regarding whether these changes occur in the early stages or are associated with well-controlled diabetes.

In our study, we observed significant differences in mean endothelial cell density (ECD) and central corneal thickness (CCT) between diabetic and non-diabetic patients. Additionally, we identified a statistically borderline association between diabetes duration and CCT.

It is important to acknowledge several limitations in this study. One of the primary weaknesses is its retrospective case-control design, rather than a prospective longitudinal study. Additionally, the study population was clinic-based rather than population-based.

Nonetheless, we anticipate that, due to the large sample size and careful adjustment for multiple potential confounders, our study will provide a comprehensive portrayal of corneal changes in the diabetic population.

## Conclusion

In conclusion, this study demonstrates that even in the early stages of well-controlled type 2 diabetes, patients exhibit measurable changes in corneal endothelial structure, including reduced endothelial cell density and increased central corneal thickness, compared to non-diabetic individuals. These findings suggest that subtle corneal alterations may begin earlier in the diabetic disease course than previously assumed, regardless of retinopathy status or glycemic indices. The presence of these early endothelial changes highlights the importance of thorough preoperative assessment and intraoperative protection of the corneal endothelium in diabetic patients undergoing ocular surgery. By identifying these subclinical alterations, ophthalmologists can better anticipate surgical risk and tailor their approach to optimize visual outcomes in this growing patient population.
